# Multimodal optical imaging and genetic features of AB variant GM2 gangliosidosis: a case report

**DOI:** 10.3389/fped.2023.1147836

**Published:** 2023-05-05

**Authors:** Qin Chen, Fang Lu

**Affiliations:** Department of Ophthalmology, West China Hospital, Sichuan University, Chengdu, China

**Keywords:** AB variant GM2 gangliosidosis, cherry-red spot, optical coherence tomography, gene, fundus fluorescein angiography (FFA)

## Abstract

**Background:**

AB variant GM2 gangliosidosis is an extremely rare autosomal recessive lysosomal storage disease. Macular cherry-red spots are the most commonly described ocular sign in this disease. Here, for the first time we report a case of an infant with AB variant GM2 gangliosidosis, along with multimodal optical imaging and genetic testing results.

**Case description:**

A 7-month-old Chinese girl presented to the hospital with nystagmus for 2 months. Her family history for this condition showed negative results, and her parents were not known to be consanguineous. Fundus photography showed a cherry-red spot with a ring of whitish infiltrate surrounding both macula. Fundus fluorescein angiography showed normal retinal circulation and vessels. Optical coherence tomography (OCT) revealed a thickening and increased reflectivity of the inner retinal layers with a shadowing effect on the outer structures. The patient had no obvious neurological symptoms, and the MRI results of the head were normal. The whole-exome genome sequencing results showed that there was a homozygous deletion (chr5: 150639196-150639548) of exon 2 in the *GM2A* gene. Finally, the patient was diagnosed with AB variant GM2 gangliosidosis.

**Conclusions:**

AB variant GM2 gangliosidosis is a rare disease affecting multiple nervous systems. Before the occurrence of typical neurological symptoms, the clinical features of fundus photography and OCT help us diagnose GM2 gangliosidosis.

## Introduction

GM2 gangliosidoses are a group of autosomal recessive lysosomal storage diseases characterized by the accumulation of glycosphingolipids in the nerve cells. According to different causative genes, GM2 gangliosidoses are divided into three subtypes: Tay–Sachs disease (*HEXA* gene), Sandhoff disease (*HEXB* gene), and AB variant GM2 (*GM2A* gene). AB variant GM2 gangliosidosis is a very rare form of disease caused by a lack of the GM2 activator protein. The typical clinical manifestations of GM2 gangliosidoses are neuromotor retardation and regression, hypotonia, and visual impairment (1). Macular cherry-red spots are the most commonly described ocular sign in GM2 gangliosidoses.

However, a full examination of optical coherence tomography (OCT) and fundus fluorescein angiography (FFA) for this specific macular abnormality has not yet been completed. Ophthalmologists can see the various layers of the retina and the vascular systems around the macula using OCT and FFA, which aids in a deeper understanding of macular illness. Here, we provide the findings of OCT, FFA, and genetic testing in a newborn with AB variant GM2 gangliosidosis.

## Case description

**Figure 1:**
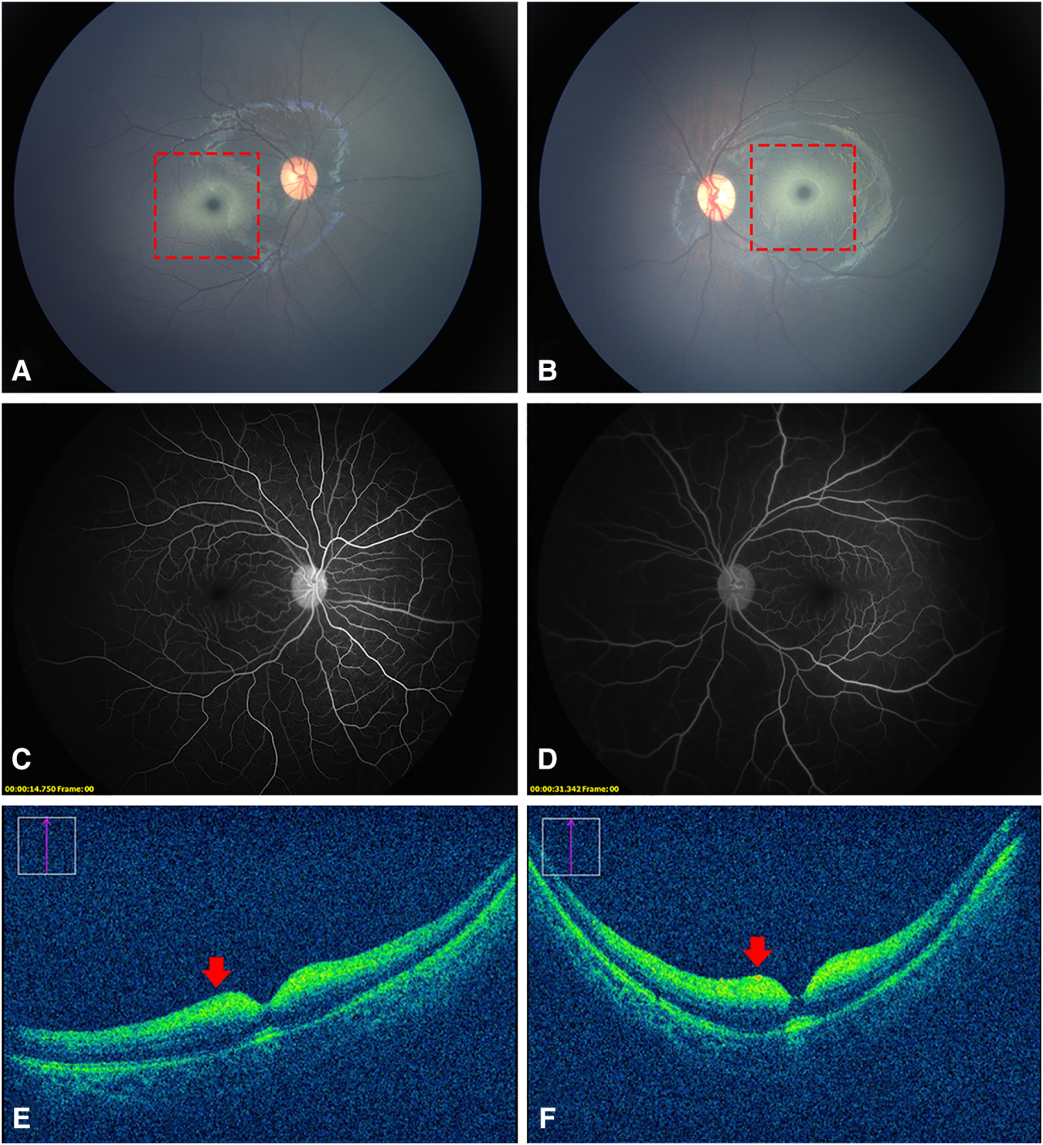
Fundus photography, FFA, and OCT images of the patient. (**A,B**), a cherry-red spot with a ring of whitish infiltrate within a 1–2 PD area surrounding the fovea (dotted red squares). (**C,D**) normal retinal vessels without obstruction. (**E,F**) thickened and increased reflectivity of the inner retinal layers with a shadowing effect on the outer structures (red arrows). It was difficult to distinguish the inner retinal structure.

A 7-month-old Chinese girl presented to the hospital with nystagmus for 2 months. The patient was born at 37 weeks’ gestation and weighed 3,400 g. Her growth and development were normal, except that she was unable to sit. Her family history was negative for this condition, and her parents were not known to be consanguineous. An examination of the anterior segment of both her eyes revealed no abnormalities. Color fundus photography (RetCam III, Natus Medical, USA) showed a cherry-red spot with a ring of whitish infiltrate surrounding both macula (Figures 1A,B). FFA showed normal retinal circulation and vessels (Figures 1C,D). OCT (OPMI LUMERA and RESCAN 700, Carl Zeiss Meditec, AG, Germany) revealed a thickening and increased reflectivity of the inner retinal layers with a shadowing effect on the outer structures. The marked thickening and hyperreflective part was within a 1–2 papillary diameter (PD) area around the fovea. It was difficult to distinguish the inner retinal structure (Figures 1E,F). The head MRI of the girl’s head revealed no visible anomalies.

**Figure 2:**
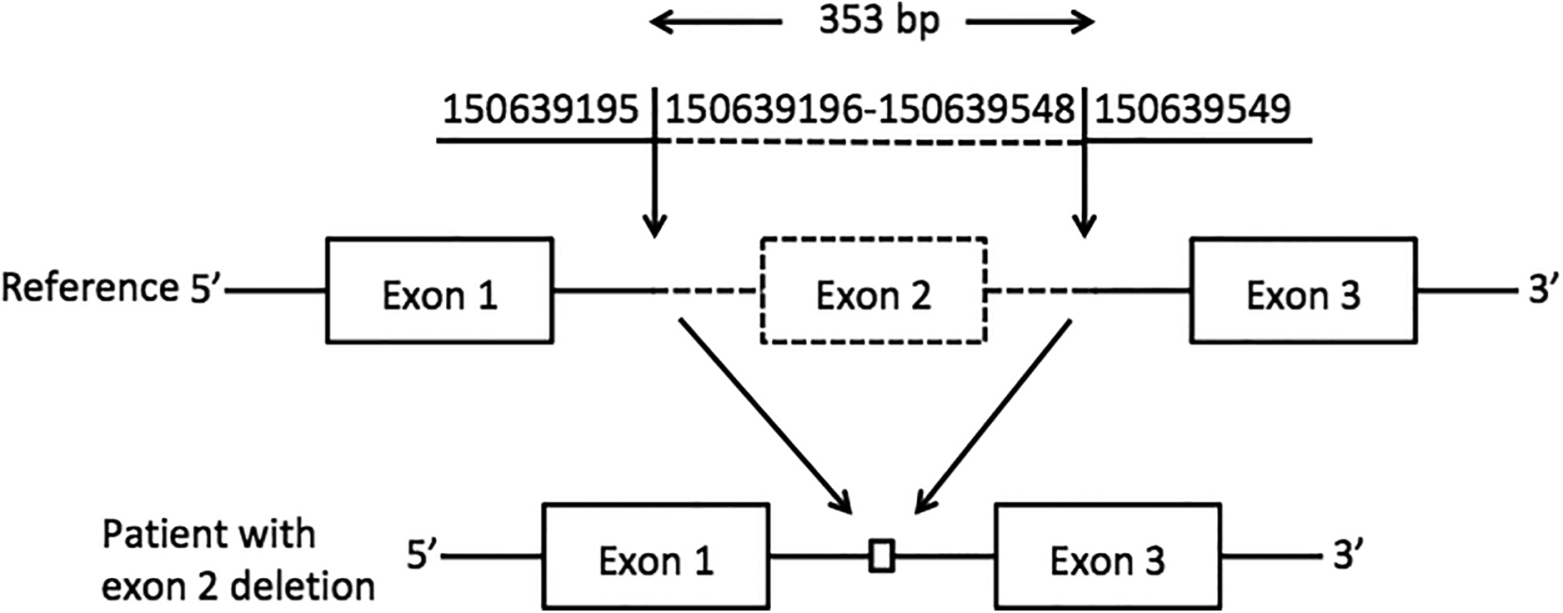
Exon 2 deletion in GM2A. A schematic representation of the deletion detected in the GM2A gene. The deleted region is represented by dotted lines, whereas the intact region is represented by solid lines. The downward arrows indicate the breakpoints of the deletion.

A novel homozygous pathogenic variant in GM2A was detected in the whole-exome genome sequencing of the patient. The sequencing results showed that there was a homozygous deletion (Chr5: 150639196-150639548) of exon 2 in the *GM2A* gene, which revealed GM2 activator protein deficiency. The parents of the patient had heterozygous mutations in the same genetic locus (Figure 2).

Finally, the patient was diagnosed with AB variant GM2 gangliosidosis. One and a half months later, more abnormal findings were reported and these were confined to the nervous system. The girl lost the ability to roll over and hold her bottle. She developed hyperacusis, a heightened sensitivity to sound, and her natural smile lessened. However, there was no effective treatment for this disease.

## Discussion

AB variant GM2 gangliosidosis is an extremely rare lysosomal storage disease caused by a deficiency of the GM2 activator protein, which makes an enzyme cofactor for beta-hexosaminidase. No more than 20 cases have been reported thus far (2–4). This disease is not easy to detect by neurologists in the early stage without obvious degenerative changes occurring in the nervous system. This is the first case report from China of AB variant GM2 gangliosidosis, and it was primarily diagnosed by the ophthalmologist. The symptoms and signs of the eyes play a very important role in the early diagnosis of the disease.

The most common clinical optical findings of AB variant GM2 gangliosidosis are cherry-red spots (72%) and nystagmus (22%) (2). Cherry-red spots, or “perifoveal white patch”, a term coined by Canadian researchers (5), are caused by the accumulation of glycolipids within the retinal ganglion cell layer of both eyes. Unlike the cherry-red spots formed by the edema of the inner retinal cells as a result of central retinal artery occlusion (CRAO), the perifoveal whitish area of GM2 gangliosidosis, in the patient in this study, was concentrated only in the range of approximately 1–2 PD around the fovea. This abnormal change corresponded to thickened hyperreflective areas on OCT images. FFA did not reveal any occlusion of the retinal vessels or late peripheral vascular leakage. All these indicated that these cherry-red spots were completely different from those resulting from CRAO. In addition to gangliosidosis, this characteristic cherry-red spot is seen in other lysosomal disorders such as Niemann–Pick, metachromatic leukodystrophy, sialidosis, and Farber disease (6).

A review of the literature revealed that this was for the first time OCT and FFA images of GM2 gangliosidosis have been obtained. Similar to other lysosomal disorders, OCT showed hyperreflective inner layers adjacent to the macula and a relative hyporeflectivity of the layers underneath (7). Different from sialidosis, the nerve fiber layer and the border between different inner retina layers was hardly recognizable in our patient. We reasoned that this unique appearance might be explained by prior histology findings. The nerve fiber layer in newborns with GM2 gangliosidosis was described in pathological research as being extremely thin. In addition to the cytoplasm of the retinal ganglion cells filled with large numbers of concentric membranous bodies, there were also a few abnormal vesicular lysosomes in the cells of the inner nuclear layer (8). Moreover, the retinal and OCT structures of infants differed from those of older kids or adults, in which the inner retina cells are more concentrated in the macular area (9). This might also be responsible for the abnormal findings of OCT in our case. Our OCT images could provide valuable clinical experience to better understand the ocular manifestations of this disease.

## Conclusions

In summary, AB variant GM2 gangliosidosis is an extremely rare type of lysosomal storage disease affecting multiple neurological systems. Before the occurrence of typical neurological symptoms, the clinical features of fundus photography and OCT help us to diagnose GM2 gangliosidosis early.

## Data Availability

The data presented in the study are deposited in the Sequence Read Archive repository, accession number PRJNA962274.
